# Effect of chamomile on chemotherapy-induced neutropenia in pediatric leukemia patients: A randomized triple-blind placebo-controlled clinical trial

**Published:** 2020

**Authors:** Babak Daneshfard, Mahdi Shahriari, Alireza Heiran, Majid Nimrouzi, Hassan Yarmohammadi

**Affiliations:** 1 *Student Research Committee, Shiraz University of Medical Sciences, Shiraz, Iran*; 2 *Essence of Parsiyan Wisdom Institute, Phytopharmaceutical Technology and Traditional Medicine Incubator, Shiraz University of Medical Sciences, Shiraz, Iran *; 3 *Hematology Research Center, Namazi Hospital, Shiraz University of Medical Sciences, Shiraz, Iran*; 4 *Department of Persian Medicine, School of Medicine, Shiraz University of Medical Sciences, Shiraz, Iran*

**Keywords:** Chamomile, Chemotherapy, Leukemia, Matricaria chamomilla, Neutropenia, Persian medicine

## Abstract

**Objective:**

Chemotherapy-induced neutropenia is one of the main side effects of acute lymphoblastic leukemia (ALL) treatment. In this trial, we evaluated the efficacy of chamomile in management of neutropenia.

**Materials and Methods:**

This randomized triple-blind placebo-controlled clinical trial was carried out in 2-18-year-old children with ALL. Participants in each group daily received 2.5 ml of either chamomile syrup or placebo syrup for a period of 30 days. Participants’ white blood cell (WBC), and absolute neutrophil count (ANC), as well as their quality of life were evaluated.

**Results:**

The study was completed with a total of 40 patients. An increasing trend of ANC was observed in the treatment group despite the decreasing trend in placebo group, which was statistically significant between the two groups (P Interaction=0.019, 95% confidence intervals=15.076–171.324). No serious side effects were reported.

**Conclusion:**

Using chamomile syrup as a complementary therapy in children with leukemia could improve their immunity (as it increased WBC) by minimizing chemotherapy-induced neutropenia.

## Introduction

Leukemia is the most common malignancy and one of the leading causes of death in childhood. Almost 77% of leukemia in children is due to the acute lymphoblastic leukemia (ALL) making it the most common type of leukemia (Kliegman et al., 2015 ▶). More than 6000 new cases of ALL and 1440 deaths due to ALL occurred in United States in 2014 (Farzaneh et al., 2016 ▶). Studies in Iran have also shown a considerable incidence rate (Rajabli et al., 2013 ▶; Zolala et al., 2004 ▶). Therapy begins with 4 weeks of “remission chemotherapy” to eradicate malignant cells in the bone marrow (Orkin et al., 2015 ▶). 

Chemotherapy-induced neutropenia as one of the most common complications of therapy, is a serious hematologic toxicity which increases the mortality and reduces the rate of response to treatment (Crawford et al., 2004 ▶; Dinan et al., 2015 ▶). Moreover, it can cause other complications such as infection, fever, and prolonged hospitalization. Granulocyte colony stimulating factor (GCSF) reduces neutropenia and the rate of infection in these patients; however, it has a high cost and may cause side effects such as splenomegaly (Barlak et al., 2004 ▶), bone pain (Gavioli and Abrams, 2017 ▶), and vasculitis (Andavolu and Logan, 1999 ▶). Therefore, any treatment approach that protects against neutropenia, reduces the need for GCSF and minimizes other complications of neutropenia, will be helpful. 

Nowadays, using complementary and alternative medicine (CAM) is being increasingly applied (Hashempur et al., 2015 ▶). Various types of these unconventional methods are used by evidence-based approaches alongside conventional medicine which is known as integrative medicine (IM) (Daneshfard et al., 2019 ▶). Herbal medicine is considered one of the most common and most popular CAM methods with a long history (Jabbari et al., 2017 ▶; Atarzadeh et al., 2017 ▶). 

*Matricaria chamomilla* L. also known as German chamomile, is an annual plant of Asteraceae family. As one of the best-known medicinal herbs, chamomile has been used in a wide variety of health products (McKay et al., 2006 ▶). Its flower essence contains α-bisabolol, flavonoids like apigenin, and azulene compounds such as chamazulene (Garcia-Pinto et al., 2013 ▶; Srivastava et al., 2009 ▶). Different studies revealed its anti-microbial (Marino et al., 2001 ▶), anti-inflammatory (Miguel, 2010 ▶), anti-oxidant (Sebai et al., 2014 ▶), and anti-cancer (Srivastava and Gupta, 2007 ▶) properties.

Anti-cancer effects of chamomile are caused by apigenin which is one of the major flavonoids that has a boosting effect on the immune system (Shukla and Gupta, 2010 ▶). Its promising effect on various cancer cells of human has made it a safe anti-cancer agent (Hosseinpour et al., 2016 ▶). For example, bisabolol oxide A in German chamomile has shown anti-proliferative effects on human leukemia K562 cells (Ogata-Ikeda et al., 2011 ▶). 

Regarding the safety of chamomile, its oral usage has been approved by German Commission E (American Botanical Council, 1998 ▶). As an immunomodulatory agent (Lee et al., 2010 ▶), it has been used as a safe natural product in pediatric patients (Godwin et al., 2013 ▶). 

Moreover, Persian medicine as a comprehensive medical system (Atarzadeh et al., 2016 ▶), has proposed chamomile for various diseases, and some of such applications were evaluated in clinical trials (Hashempur et al., 2017 ▶; Shoara et al., 2015 ▶; Sharifi et al., 2017 ▶). As a booster of *Hararat-e-Gharizi* (innate heat), this herb has an endurance strengthening effect that is very useful for fatigue and body pain (common symptoms observed in leukemic patients) (Qarshi, 2008 ▶; Ibn-Sina, 2005 ▶). Accordingly, we assumed that chamomile may have a boosting effect on weakened immune system. 

Although there is a growing trend toward the use of herbs for cancer support, few clinical studies were done to investigate the effect of herbal therapy in children with cancer. In this study, we aimed at evaluating the effect of chamomile syrup as a complementary therapy on chemotherapy-induced neutropenia in pediatric ALL patients. 

## Materials and Methods

**Study design**

This randomized, placebo-controlled, clinical trial was conducted with 1:1 allocation ratio and designed as a triple-blinded study: the principal researcher, participants and the statistician who was responsible for data analysis were blinded to the allocation groups. The study protocol remained unchanged until the end of the trial.

**Ethical considerations**

The local Research Ethics Committee of Shiraz University of Medical Sciences (SUMS) approved the proposal of this trial (research ID: IR.SUMS.REC.1396.11). The ethical guidelines of Helsinki Declaration were observed in this trial. In addition, we registered the proposal in Iranian Registry of Clinical Trials (IRCT.ir; registration No. IRCT2013021612486N1). As the participants were under the legal age, their parents signed the written informed consent form. 

**Preparation of drug and placebo**

German chamomile flower was purchased from a natural farm in Tehran. The purchased herb was evaluated by a botanist (Miss. Sedigheh Khademian) using powder microscopy method in the Herbarium Center of School of Pharmacy, SUMS. The identity was confirmed as *Matricaria chamomilla* L. (Voucher No. PM1089). Then, the herbs were washed, disinfected, and dried at 30^o^C. Dried herb was crushed using an electric blade. Subsequently, maceration extraction method was applied using ethanol 70% with an herb-to-solvent ratio of 1:5. The obtained extract was dried in a rotary dryer with the pressure of 22-26 mmHg at 50^o^C. This ethanol extract was used for preparing chamomile syrup in a way that any milliliter of the syrup contained 50 mg of chamomile extract. The placebo contained syrup base without chamomile extract. Orange flavor was added to both drug and placebo syrups in order to hide the taste and flavor. In addition, it improved the taste and made the syrups more suitable for children.

**Apigenin determination by high-performance liquid chromatography (HPLC) method**

Apigenin, as the main flavonoid content of chamomile, was quantified using HPLC analysis. This method was applied according to the United States Pharmacopeia and National Formulary 2018 (Convention USP, 2017 ▶). It was performed with the following features in reversed-phase chromatography (RPC): ultraviolet (UV) detector wavelength: 335 nm, flow rate: 1 ml/min, and injection volume: 15 µl. Mono potassium phosphate (0.005 molar solution; pH 2.55±0.05) and acetonitrile plus ethanol (13:7 molar ratio) solutions were also used in gradient elution mode of mobile phase. A concentration of 25 µg/ml of standard sample was prepared in water-methanol solution with 1:1 ratio. Syrup sample was injected to the apparatus after filtration through a 0.45 µm filter. 

**Figure 1:**
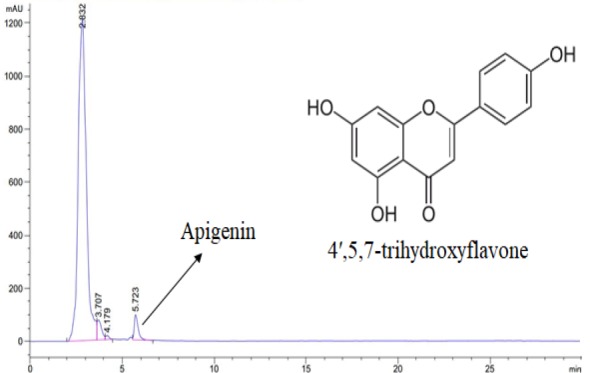
HPLC-chromatogram of the drug sample showing apigenin peak with the retention time of 5.723 minutes (mAU reflects milli absorption units in Y-axis)

Figure 1 shows the HPLC-chromatogram of pharmaceutical sample that represents apigenin peak (retention time: 5.723 min). Comparison between drug and standard sample revealed that apigenin concentration was equal to 1.27 mg/ml in drug syrup.

**Microbiologic tests**

A sample of the prepared drug was microbiologically evaluated according to United States Pharmacopeia 41 (USP 41) (Convention USP, 2017 ▶). All of the following tests: mesophilic aerobic bacteria, *Pseudomonas aeruginosa*, *Escherichia coli*, *Salmonella*, *Shigella*, *Bacillus cereus*, *Clostridium botulinum*, *Staphylococcus aureus*, mold, and yeast, were in accordance with the reference values all in 1 gram (document code: F137-01-25).

**Inclusion and exclusion criteria**

In this study, all new cases of pediatric leukemia patients who were admitted to Shiraz Amir Oncology Hospital (affiliated with SUMS) were evaluated from May to December 2017. These patients were admitted for the first time to receive “remission chemotherapy” as their initial phase of treatment which is known as “induction of remission”. Based on the results of flow cytometry confirmed by a pathologist (a faculty member of SUMS pathology department), those with the diagnosis of B-cell acute lymphoblastic leukemia in the age range of 2-18 years, were included when the informed consent was signed by their parents. Those who had sensitivity to chamomile or refused to participate, were excluded. 

**Randomization, blinding and concealment of allocation**

Computer-generated block-randomization with equal blocks (block size 4) was used blindly to create a block-randomization list for two parallel groups (A and B sequences) by the statistician. Based on the sequence number of the list, each patient was assigned to one of the groups. Allocation was kept confidential for patients themselves, researcher, and the statistician. Moreover, taste, smell, color, and the container of drug and placebo were the same between the groups in order to keep the allocation hidden until the end of the study. Two sets of computer-generated barcodes were assigned to each group’s bottles, and uncovered only after completing the data analysis. 

**Intervention**

Participants in each group daily received 2.5 ml of either drug (containing 125 mg of chamomile extract) or placebo syrup for the period of 30 days. A graded spoon was given to the parents for proper administration of the prescribed dose of syrup. They also store the syrup in the refrigerator after opening the bottle cap. This intervention was initiated within the first 24 hours of remission chemotherapy without any change in routine treatment strategies of the patients including GCSF injection. We followed the registered patients during their hospital course until their first outpatient clinic follow-up after the end of intervention.

**Data gathering**

Demographic data was recorded after the enrollment of eligible patients. Then, TNO-AZL Preschool Children Quality of Life (TAPQOL) questionnaire was used for evaluating the quality of life (QoL). This questionnaire has 43 questions evaluating 12 items in four domains of physical, cognitive, social, and emotional functioning during the past three months (adjustable time in different studies) (Rahimi et al., 2014 ▶). As the preschool children cannot complete the form, it is done by their parents. The parents were also asked for filling a form indicating their compliance during the study (number of days that syrup has been given to the patients). Lab data was recorded during the hospital course. After the end of the intervention, patients were evaluated regarding their QoL, lab data, and compliance in outpatient clinic follow-up. The principal investigator collected all the data (B. Daneshfard). 

**Outcome measures**

Primary outcome measures were white blood cell (WBC) count and absolute neutrophil count (ANC) which were repetitively measured during and after the hospital course. Quality of life assessed before and after the intervention and other lab data including, platelet count, and hemoglobin, aspartate transaminase, alanine transaminase, alkaline phosphatase, and lactate dehydrogenase levels were considered secondary outcome measures.

**Statistical analysis**

Statistical analysis was carried out using statistical package for social sciences (SPSS) (IBM Corp. Released 2013, IBM SPSS statistics for windows, version 22.0. Armonk, NY: IBM Corp.) and R programming language (version 3.4.4 for windows). Qualitative and quantitative variables were described using frequency (percent) and mean±standard deviation (SD), respectively. Baseline characteristics were compared between the groups using the Chi-square test for qualitative variables and the Wilcoxon rank-sum test for quantitative variables. Over the course of therapy, generalized estimating equation (GEE) models were applied to model repeated measurement of WBC, ANC, and other laboratory measurements. Regarding the heterogeneity in the arrival (due to the excessed blast in peripheral blood of WBC), the day at which WBC/ANC was minimum reflective of the lymphoblastic suppression by chemotherapy, was considered the initial analysis day in order to normalize the base values of WBC and ANC; this day was the 8th day of the study for both variables. Thence, multiple imputation method was applied to control the influence of missing data. This method was implemented in the Amelia II package (version 1.7.5) of R that was superior to the commonly implemented last observation carried forward (LOCF) methods. A p-value≤0.5 was considered statistically significant.

## Results

**Study flow and baseline characteristics**

**Figure 2:**
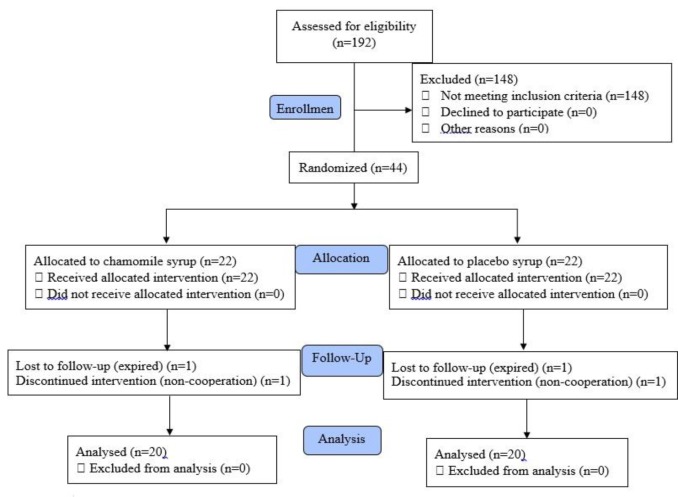
The consort flow diagram of the study

A total number of 192 leukemic patients were evaluated for eligibility criteria, among which 44 were enrolled and 40 patients completed the study. The study flow is shown in Figure 2. Table 1 shows demographic, baseline characteristics and laboratory data; the two groups showed no statistical difference in all variables except for aspartate transaminase level. Such result was also found for positive blood culture, positive typhilitis, hospitalization time, compliance to assigned treatment and GCSF injection during hospital course. 

**ANC and WBC**

**Figure 3:**
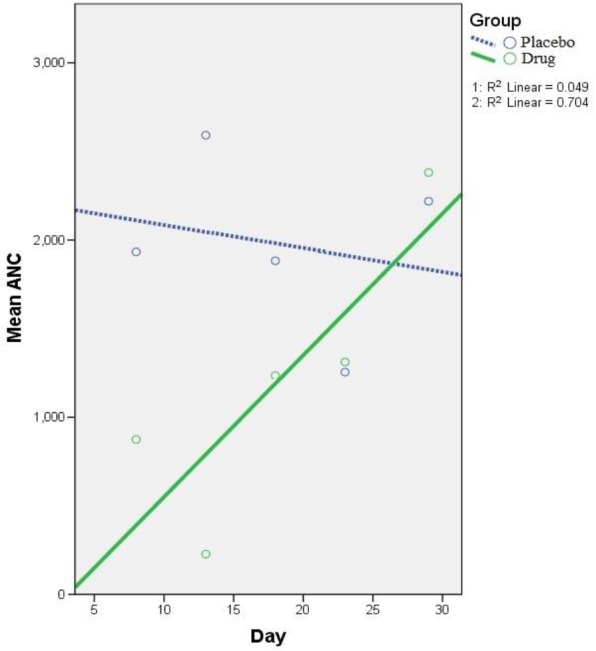
Generalized estimating equation (GEE) parameter estimates of absolute neutrophil count (ANC) repeated measurements (5-day intervals) in drug and placebo groups [8th-to-28th day] – Intercept: 2216.72, Standard error of mean (SEM): ±553.76; β coefficient - P Reference (placebo group): -13.21 (SEM: ±739.89) - 0.661; β coefficient - P Interaction: 93.2 (SEM: ±39.86) - 0.019. P Reference group (placebo group) indicates if the change on ANC trend in placebo group is significant or not. P Interaction contrasts the slope of linear trend on ANC between drug group and placebo group

**Figure 4:**
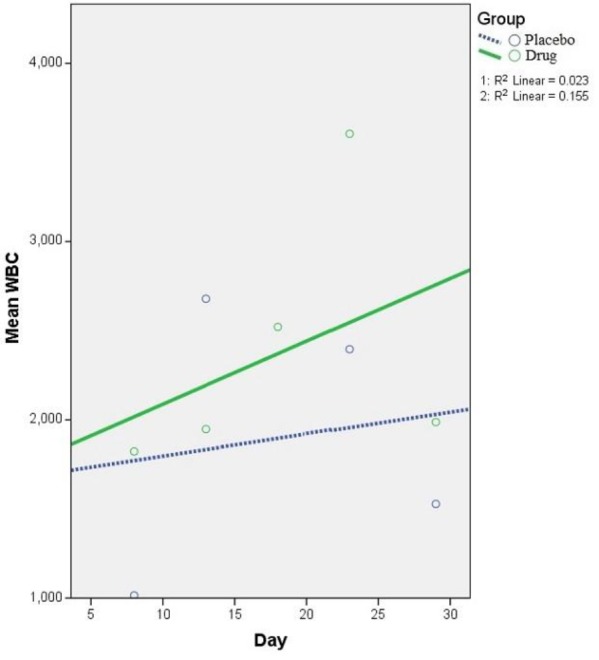
Generalized estimating equation (GEE) parameter estimates of white blood cell (WBC) count repeated measurements (5-day intervals) in drug and placebo groups [8th-to-28th day] – Intercept: 1672.498, Standard error of mean (SEM): ±1672.5; β coefficient - P Reference group: 12.35 (SEM: ±1074.93) - 0.737; β coefficient - P Interaction: 22.91 (SEM: ±58.56) - 0.696. P Reference (placebo) group indicates if the change on WBC trend in placebo group is significant or not. P Interaction contrasts the slope of linear trend on WBC between drug group and placebo group

Figures 3 and 4 illustrate the trend of ANC and WBC changes in drug and placebo groups, respectively. An increasing trend for ANC was seen in drug group despite decreasing trend in placebo group. The result showed a higher 5-day rate of ANC rise in drug group (79.86 vs. -13.21 for drug and placebo groups, respectively); this was faster in comparison with placebo group (difference in slopes: P interaction=0.019, 95% confidence intervals=15.076–171.324). Despite the higher WBC increase rate in drug group, this was not statistically significant (35.26 vs. 12.35; P interaction=0.696, 95% confidence intervals=-91.869–137.692). 

**Other laboratory data**

Table 2 shows the GEE output of other six laboratory measurements. In contrast to placebo group, the platelet count slope was positive in drug group (1638.52 vs. -140.59). Both groups showed a slight decreasing trend for hemoglobin (-0.04 vs. -0.02). However, that was significant neither for platelet count (P interaction=0.116, 95% confidence intervals=-442.021–4000.242) nor hemoglobin (P interaction=0.873, 95% confidence intervals=-0.025–0.021). No statistical significance was observed in trend of other blood markers changes.

**Table 1:** Demographic and baseline characteristics and laboratory data of drug and placebo groups

**Characteristics**	**Drug (n=20)**	**Placebo (n=20)**	**p-value** ^ a^	**Total (n=40)**
Male sex [n (%)]	14 (70)	11 (55)	0.327	25 (62.5)
Age [year, mean±SD]	6.39±5.04	5.85±3.98	0.534	6.12±4.49
Body surface area [m^2^, mean±SD]	0.85±0.37	0.8±0.36	0.819	0.83±0.36
Fars ethnicity	15 (75)	14 (70)	0.668	29 (72.5)
WBC^ b^ [10^3 ^cells/µl, mean±SD]	7.3±4.83	10.51±10.12	0.561	8.9±7.99
ANC^ b^ [10^3 ^cells/µl, mean±SD]	0.5±0.5	1.08±1.54	0.543	0.79±1.17
Plt^ b^ [10^3^ cells/µl, mean±SD]	127.7±122.3	82.8±97.41	0.18	105.3±111.5
Hb^ b^ [g/dl, mean±SD]	8.77±2.05	8.38±2.16	0.499	8.58±2.09
AST^ b^ [IU/l, mean±SD]	23.52±36.93	46.65±38.51	0.014	35.09±39.04
ALT^ b^ [IU/l, mean±SD]	43.39±54.52	41.9±57.16	0.756	42.64±55.14
Alk. Pho.^ b^ [IU/l, mean±SD]	304.29±120	328.65±125.09	0.659	316.47±121.6
LDH^ b^ [IU/l, mean±SD]	579.8±411.76	1010.15±891.16	0.086	795±719.01
Positive blood culture ^c^	2 (10)	2 (10)	1	4 (10)
Positive typhilitis ^c^	0	0	-	0
GCSF injection ^c^	5 (25)	5 (25)	1	10 (25)
Hospitalization time [day, mean±SD]	23±4.75	24.2±7.47	0.626	23.6±6.21
Compliance [day, mean±SD]	26.8±4.93	26.3±4	0.378	26.57±4.44

^a^ Wilcoxon signed rank test or Chi-square test; ^b ^Multiple imputation was used; ^c^ recorded during hospital course; Abbreviations: SD: standard deviation, WBC: white blood cell, ANC: absolute neutrophil count, Plt: platelet, Hb: hemoglobin, AST: aspartate transaminase, ALT: alanine transaminase, Alk. Pho.: alkaline phosphatase, LDH: lactate dehydrogenase, GCSF: granulocyte colony stimulating factor

**Table 2:** GEE output of other laboratory measurements^ a ^[8^th^-to-28^th^ day]

	**Reference group (placebo group)**		**Interaction**	
	**β coefficient**	**p-value**	**β coefficient**	**p-value**
**Plt**	-140.59	0.882	1779.11	0.116
**Hb**	-0.02	0.835	-0.02	0.873
**AST**	-0.38	0.026	0.24	0.412
**ALT**	-0.39	0.604	0.88	0.434
**Alk. Pho.**	0.328	0.815	-0.06	0.973
**LDH**	-0.06	0.977	0.308	0.925

Abbreviations: Plt: platelet, Hb: hemoglobin, AST: aspartate transaminase, ALT: alanine transaminase, Alk. Pho.: alkaline phosphatase, LDH: lactate dehydrogenase. P Reference group (placebo group) indicates if the change on the variable trend in placebo group is significant or not. P Interaction contrasts the slope of linear trend on variable between drug and placebo groups.

**Quality of life**

Table 3 depicts before-after treatment TAPQOL scores. Comparing baseline score with after-treatment TAPQOL score, no significant change was observed separately regarding physical functioning, social functioning, cognitive functioning, emotional functioning, and total score in both groups. Also, this comparison showed no significant difference between the two groups. However, both groups showed significant improvements in appetite and a subdomain of physical functioning (drug group: p=0.003; control group: p=0.002). It is worthy to note that skin related QoL score was significantly decreased in drug group (p=0.004). 

**Table 3:** Comparing before-after treatment TAPQOL score

**Domain**	**Before treatment ** **[mean±SD** ^ a^ **]**	**After treatment** **[mean±SD]**	**p-value ** ^b^
** Drug group**			
Physical functioning Sleeping Appetite Lungs Stomach Skin Motor functioning	79.75±10.17 ^c^ 68.12±20.77 60.42±19.28 93.75±13.48 78.75±22.37 97.92±5.32 82.5±25.7	81.44±15.23 78.12±22.9 87.92±21.37 93.33±13.94 73.75±23.61 85±19.04 74.06±25.75	0.629 0.058 0.003 1 0.536 0.004 0.420
Social functioning Social functioning Problem behavior	63.25±15.92 85.83±24.94 53.57±16.63	65.75±19.08 80±27.36 59.64±22.38	1 0.203 0.469
Cognitive functioning Communication	97.81±5.08	98.44±5.69	0.672
Emotional functioning Anxiety Positive mood Liveliness	70±18.24 58.33±25.65 75±22.62 76.67±28.82	61.67±20.14 56.67±21.22 69.17±27.19 59.17±23.86	0.162 0.704 0.447 0.050
Total	78.13±8.97	78.47±13.38	0.931
** Control group**			
Physical functioning Sleeping Appetite Lungs Stomach Skin Motor functioning	72.12±16.93 68.12±27.35 52.5±26.91 88.75±19.92 73.75±25.69 86.67±19.94 66.25±34.5	81.56±11.85 78.75±24.2 88.75±24.82 86.25±22.5 85±15.67 91.25±9.92 65.56±11.85	0.062 0.207 0.002 0.524 0.162 0.594 1
Social functioning Social functioning Problem behavior	63.75±23.39 82.5±29.36 55.71±28.25	63.25±20.6 70.83±33.71 60±25.32	0.983 0.165 0.507
Cognitive functioning Communication	97.5±6.54	89.37±23.31	0.141
Emotional functioning Anxiety Positive mood Liveliness	65±22.66 56.67±30.3 72.5±25.52 65.83±30.81	63.61±18.77 59.17±23.24 70±22.03 61.67±26.55	0.743 0.622 0.703 0.671
Total	72.95±14.59	77.35±11.77	0.198

^a ^SD=standard deviation; ^b^ Wilcoxon signed rank test; ^c^ crude domains scores are transformed to a 0–100 scale.

## Discussion

The goal of this study was to evaluate the effect of chamomile syrup on chemotherapy-induced neutropenia in pediatric leukemia patients. Based on our findings, using chamomile syrup as a complementary therapy improved the immunity (i.e. increased WBC count) in leukemia patients undergoing chemotherapy by reducing their neutropenia. 

Although nausea and vomiting are among common complications of chemotherapy, this syrup was well tolerated by children due to its good taste

In addition to its positive effect on neutrophil count, it seems that this integrative approach had no negative effect on the routine chemotherapy regime of the patients. 

Increasing trend for ANC in drug group (unlike the decreasing trend in placebo group) obviously showed the protective effect of chamomile against neutropenia, in which was also indicated by previous studies. The immunity-enhancing property of chamomile has made it a suitable choice for management of chemotherapy-induced oral mucositis (Pourdeghatkar et al., 2017 ▶; Mazokopakis et al., 2005 ▶). It is worth mentioning that oral mucositis had a linear relationship with granulocyte count and there was a coincidence of neutrophil recovery at the time of its resolution (Köstler, 2001 ▶). Additionally, an animal study revealed reactive oxygen species (ROS) scavenging properties of chamomile extract which played an important role in protecting neutrophils and erythrocytes from oxidative stress (Jabri et al., 2016 ▶). This effect explained how chamomile extract increased the neutrophil count. 

Although WBC and platelet count in drug group was increased in our study, this finding was not statistically significant which may be due to the study limitations. Evaluating this effect of chamomile could be considered in further studies. 

As shown in Table 3, our results did not show a significant change in QoL (except for skin related QoL). According to recently published systematic reviews (Fardell et al., 2017 ▶; Vetsch et al., 2018 ▶), it seems that the actual effect of therapy in childhood leukemia related QoL could be detected more accurately by the pass of time; hence, prospective studies with a longer follow-up are essential for exact QoL evaluation. 

Increasing appetite (based on the TAPQOL scores) in both groups seemed to be the result of steroid therapy as a part of their chemotherapy regimen; thus, the effect of chamomile could not be evaluated in this regard. The decrease in the skin related QoL score of drug group also seemed to be due to the dry skin. Although it was mild in severity, but this complication should be observed in future studies. 

Several constituents in chamomile account for its various therapeutic effects. As one of the important factors of chamomile antioxidant power, chamazulene has shown radical scavenging and leukotriene B4 inhibiting effects (Capuzzo et al., 2014 ▶; Safayhi et al., 1994 ▶). This aromatic azulene compound prevented lipid peroxidation as a chemo-preventive and anticancer agent (Ornano et al., 2013 ▶). It is a sesquiterpene which is responsible for the dark blue color of chamomile essential oil (Sashidhara et al., 2006 ▶). 

Apigenin is also one of the main flavones of chamomile possessing anticancer effects (Srivastava et al., 2017 ▶). It is known as a chemotherapeutic agent that causes cell-cycle arrest and autophagy in leukemic cells (Ruela-de-Sousa et al., 2011 ▶). In an animal model, apigenin has shown protective effect on aflatoxin B1-induced immunotoxicity (Choi et al., 2010 ▶). Considering its antioxidant and anti-inflammatory properties, apigenin has been known as a promising agent for cancer prevention (Shukla and Gupta, 2010 ▶). 

Finally, chamomile as an immunomodulatory herb had a boosting effect on the immune system in the immunocompromised state. This property, confirmed by current evidence, was not very far from Persian medicine literature introducing this medicinal herb as a tonic agent. 

Except small sample size and short-term follow-up of the patients, there were some limitations in this study. Blood sampling of the patients was done through a routine procedure of the hospital and we were not able (due to the ethical concerns) to force them for another fixed schedule of sampling. Moreover, we did not separate standard and high risk patients in the groups. Application of TAPQOL questionnaire was better to be done after a 3-month follow-up but we did it within a month due to the difficulties in accessibility to the patients. In addition, this questionnaire has been designed for pre-school children; however, it was also used for older children. 

Based on the results of this study, oral application of chamomile is effective in enhancing the immunity (i.e. increasing WBC count) in ALL patients. It has a protective effect against chemotherapy-induced neutropenia which makes it a safe complementary medicine in these patients. However, this property should be evaluated in further clinical evaluations with larger sample sizes and longer duration of follow-up. 
